# Screening and validation of reference genes for qRT-PCR of bovine skeletal muscle-derived satellite cells

**DOI:** 10.1038/s41598-022-09476-3

**Published:** 2022-04-05

**Authors:** Guo-Hua Wang, Cheng-Cheng Liang, Bing-Zhi Li, Xin-Ze Du, Wen-Zhen Zhang, Gong Cheng, Lin-Sen Zan

**Affiliations:** 1 College of Animal Science and Technology, Northwest A&F University, Yangling, 712100 Shaanxi China; 2 College of Animal Science and Technology, Hebei North University, Zhangjiakou, 075000 Hebei China; 3 National Beef Cattle Improvement Center, Northwest A&F University, Yangling, 712100 Shaanxi China

**Keywords:** Biotechnology, Cell biology

## Abstract

The accuracy of sixteen commonly used internal reference genes was assessed in skeletal muscle-derived satellite cells of Qinchuan cattle at different stages of proliferation and induction of differentiation to determine the most suitable ones. Quantitative real-time PCR and three commonly used algorithmic programs, GeNorm, NormFinder and BestKeeper, were used to evaluate the stability of expression of the candidate internal reference genes (*GAPDH, ACTB, PPIA, LRP10, HPRT1, YWHAZ*, *B2M*, *TBP, EIF3K , RPS9, UXT, 18S rRNA, RPLP0*, *MARVELD*, *EMD* and *RPS15A*) in skeletal muscle-derived satellite cells at 0, 12, 24, 36 and 48 h of growth and after differentiation for 0, 2, 4, 6 and 8 days. The expression of two satellite cell marker genes, *CCNA2* and *MYF5*, was used for validation analysis. The results of the software analyses showed that *GAPDH* and *RPS15A* were the most stable reference gene combinations during in vitro proliferation of bovine skeletal muscle-derived satellite cells, *RPS15A* and *RPS9* were the most stable reference gene combinations during in vitro induction of differentiation of the cells, and *PPIA* was the least stable reference gene during proliferation and differentiation and was not recommended. This study lays the foundation for the selection of reference genes for qRT-PCR during the proliferation and induction of differentiation of bovine skeletal muscle-derived satellite cells.

## Introduction

The quantity and quality of livestock skeletal muscle are key factors in determining the quality of meat and are critical factors in the management of animal husbandry^1^. The process of growth and development of skeletal muscle is complex. It requires proliferation, differentiation and fusion of myoblasts into muscle fibers, and involves a large number of changes in gene expression^2,3^. Since it is difficult to study muscle cell proliferation and differentiation in vivo, primary cultured myoblasts are commonly used as a model because of their excellent species specificity. Studying the proliferation and differentiation of bovine skeletal muscle-derived satellite cells in vitro is of great importance in animal husbandry and the food industry. The quantitative real-time PCR (qRT-PCR) method is a widely used technique for measuring relative gene expression with high throughput, accuracy, sensitivity and reproducibility^4^. It is used in a quantitative way to evaluate relative gene expression, but the accuracy of qRT-PCR results depends on the stability of the reference gene^5^. Ideally, the genes selected for reference should show similar mRNA levels at different developmental stages of an organism, and different tissues or cells should not vary due to environmental factors or bioassay treatments^6^. However, different species, different tissues of the same species, different cells of the same tissue, different stages of development of the same cells and different experimental treatments can all affect the expression of reference genes^7^. There is no universal reference gene that can be used for all cell and tissue types^8^, and choosing an inappropriate reference gene may yield inaccurate results and erroneous conclusions. Therefore, the optimal reference gene should be carefully determined for each organism and experiment. Although many studies have investigated changes in gene expression in the proliferation and differentiation of bovine skeletal muscle-derived satellite cells (bovine SMSCs), few have assessed the stability, suitability and reliability of reference genes for qRT-PCR standardization. Evidence has shown that expression of the traditional internal reference genes sometimes changes significantly under different experimental conditions^7^. The reference genes commonly used to study the development of SMSCs are β-actin (*ACTB*), 3-phosphoglyceraldehyde dehydrogenase (*GAPDH*) and 18 s ribosomal RNA (*18 s rRNA*), but they may not be ideal and reliable^9,10^. The selection of a suitable reference gene is crucial in qRT-PCR studies for accuracy of the calculations of target gene expression.

In this study, three commonly used algorithms, GeNorm^11^, NormFinder^12^ and BestKeeper^13^, were used to identify and validate the expression stability of sixteen candidate reference genes commonly used for qRT-PCR, to provide a better understanding of the proliferation and differentiation of bovine primary skeletal muscle cell models in vitro.

## Results

### Proliferation and differentiation of bovine SMSCs in vitro

**Figure 1:**
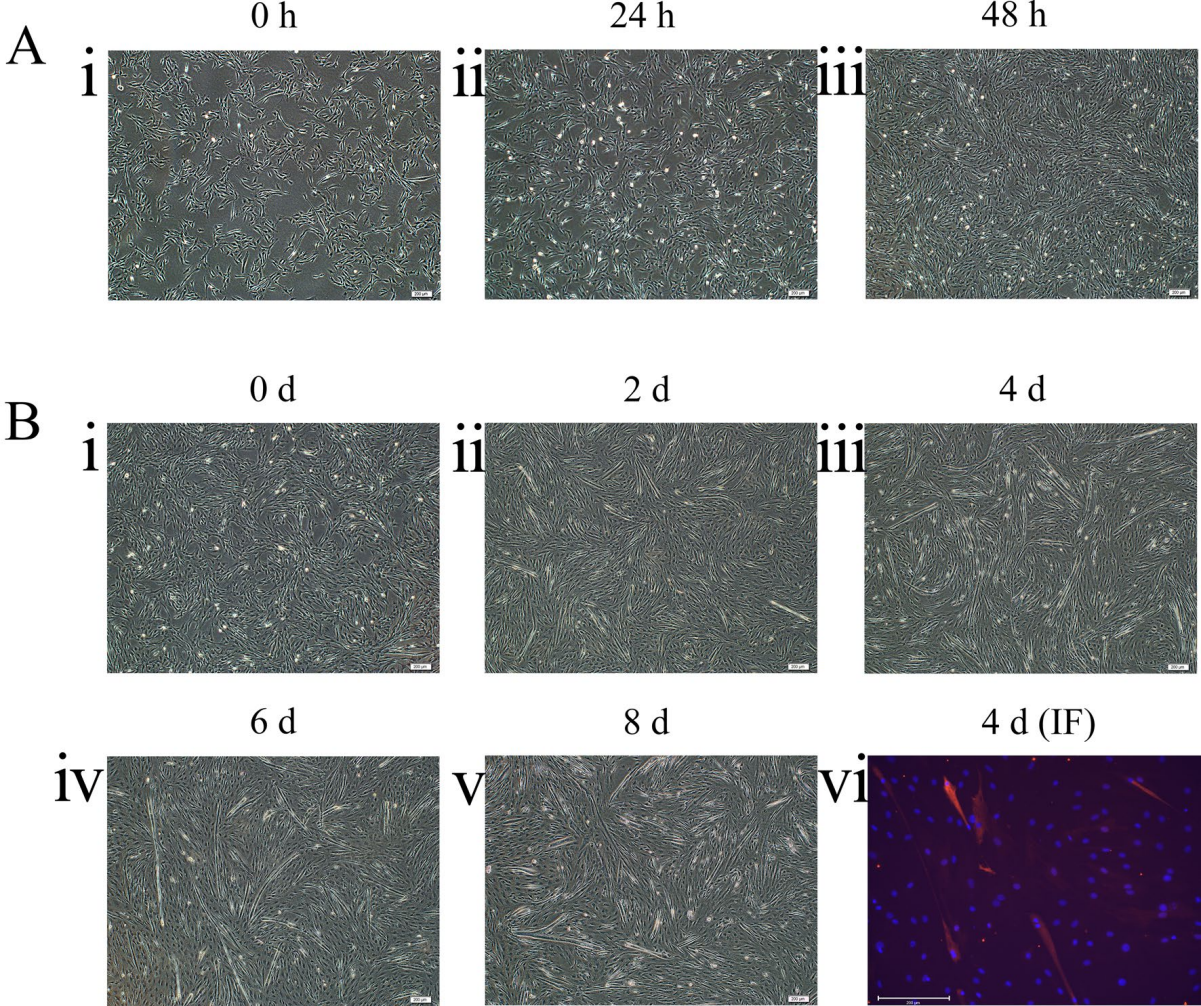
Proliferating and differentiating bovine skeletal muscle-derived satellite cells in vitro. (**A**) Cell proliferation at 0, 24 and 48 h (400 ×). (**B**-i~v) Cells at 0, 2, 4, 6 and 8 days of induced differentiation (40 ×). (**B**-vi) immunofluorescence, DAPI staining is shown in blue and MYHC is shown in red (100 ×).

Accurately determining how well bovine SMSCs proliferated and differentiated into myotubes in vitro was one goal of this study. As shown in Fig. 1A, skeletal muscle cell cultures at a confluence of about 50% were defined as 0 h to measure proliferation. The morphology of SMSCs was normal at 0 h, with a distinctive shuttle shape and good ductility. As time progressed, the number of skeletal muscle cells increased significantly with no significant morphological changes, and, by 48 h, there was local contact inhibition and increased cell polarity. This indicated that the skeletal muscle cells were in a good proliferative state and could be used for subsequent experiments. As shown in Fig. 1B, day zero (d 0) for determining differentiation of skeletal muscle cells was defined as about 80% confluence. The SMSCs in differentiation medium exhibited normal morphology at d 0, but myotubes began to appear by d 2 under differentiation induction. By d 8, most of the skeletal muscle cells had fully differentiated and fused into mature myotubes, and the length of the myotubes increased significantly. As shown in Fig. 1B(4d) (IF), immunofluorescence was performed with myosin heavy chain (MYHC) antibody, and the observed myotubes displayed strong fluorescence. This indicated that they were differentiated from myocytes and that the skeletal muscle cells used in this study had good myogenic differentiation potential and could be used in subsequent experiments.

### Primer specificity and amplification efficiency

qRT-PCR amplification of candidate reference genes and gel electrophoresis showed primer-specific amplification of the target genes (Figure S1), and fusion curve analysis also demonstrated the specificity of the primers (Figure S2).The standard curves of the candidate internal reference genes obtained by fivefold dilution gradient were linear, with amplification efficiency ranging from 90.4% to 120.8% and coefficients of determination (R^2^) ranging from 0.969 to 1.000, indicating that the primers worked well under the qRT-PCR amplification conditions and yielded accurate and reliable results.

### qRT-PCR Ct values of reference genes

**Figure 2:**
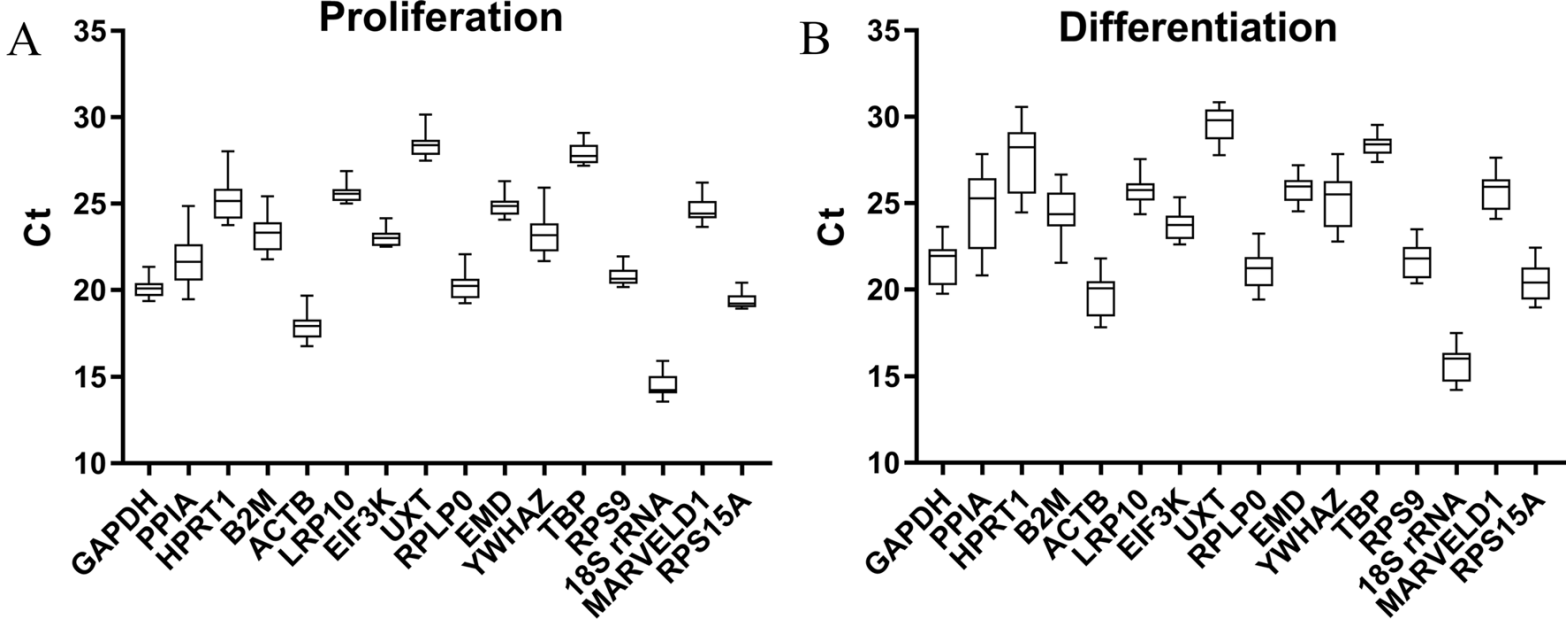
Ct value profiles of sixteen candidate reference genes in bovine SMSCs under two experimental conditions. (**A**) Ct values for proliferation. (**B**) Ct values for induced differentiation.

Ct values showed the expression levels of the candidate reference genes in proliferating and differentiating bovine SMSCs (Fig. 2). Higher Ct values indicate lower expression levels, and significant differences in expression between genes were found. The means of the Ct values of the candidate reference genes in SMSCs at different stages of proliferation and induced differentiation ranged from 13.58 to 30.16 and 14.23 to 30.85, respectively. Minimal differences in *RPS15A* expression in the proliferation stage and in *TBP* expression in the differentiation stage were observed. The highest expression of 18 s rRNA and the lowest expression of *UXT* were observed in the proliferation and differentiation stages, and *PPIA* showed the greatest variation. The statistical comparison of Ct values showed that *PPIA* was the least stable reference gene.

### Stability analysis of internal reference genes

#### GeNorm analysis

**Figure 3:**
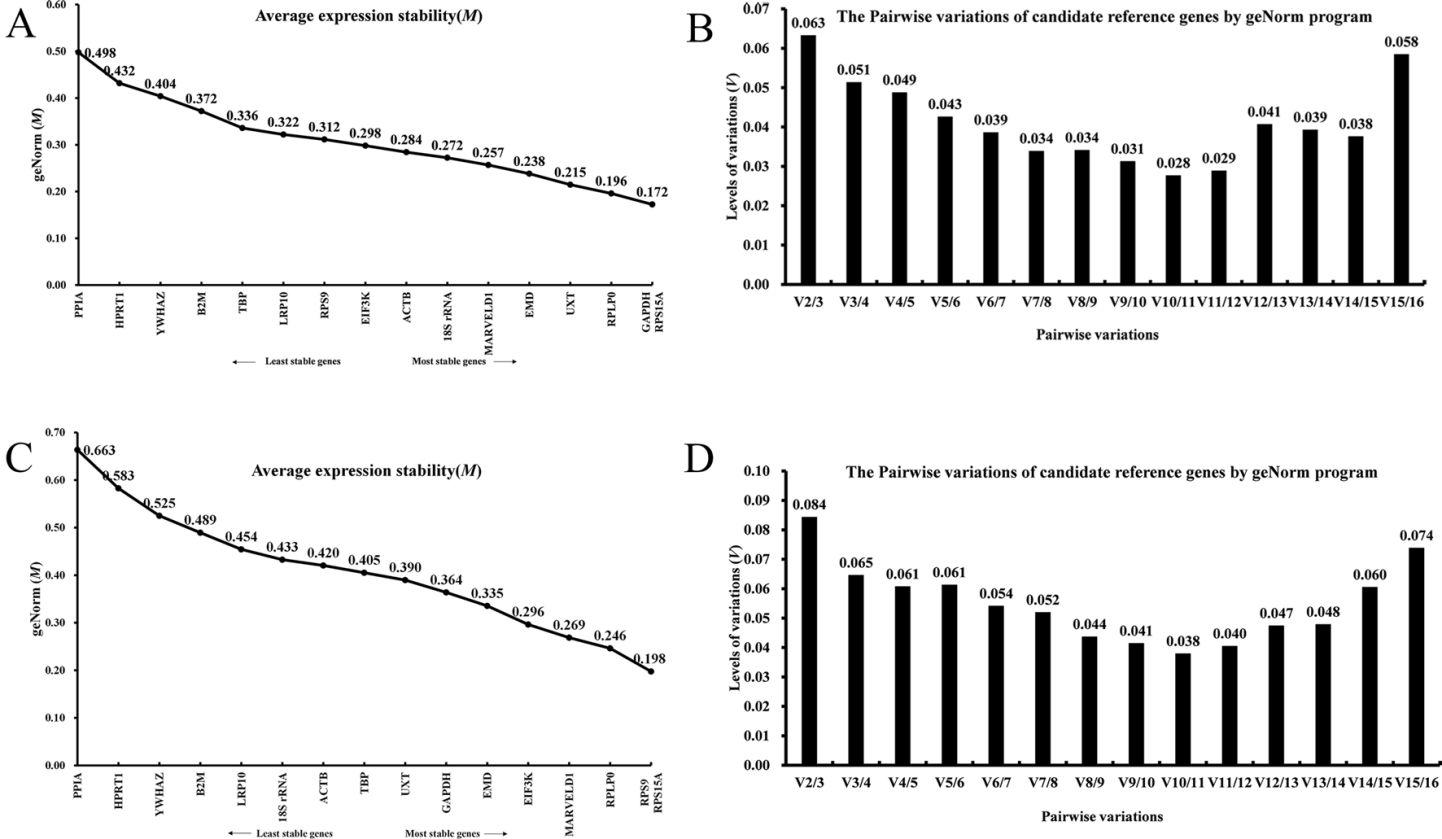
Expression stability and optimal reference genes among the candidate reference genes were determined using the GeNorm program. (**A**) Stability of candidate reference genes during proliferation of bovine SMSCs. (**B**) Optimal reference genes for proliferation of SMSCs. (**C**) Stability of candidate reference genes during differentiation of bovine SMSCs. (**D**) Optimal reference genes for use during in vitro induction of differentiation of SMSCs.

The expression stability value (M, Fig. 3) of each candidate gene was calculated by GeNorm based on the pairwise variation of the test genes, and a smaller M value equates to greater stability. The results show that all the reference genes had high stability; M values ranged from 0.172 to 0.498 during proliferation and from 0.198 to 0.663 during differentiation of bovine SMSCs. The two most stable internal reference genes were *GAPDH* and *RPS15A* for proliferation (Fig. 3A), and the two most stable internal reference genes for differentiation were *RPS15A* and *RPS9* (Fig. 3C). The software considered *PPIA* as the least stable reference gene after analysis during proliferation and differentiation (Fig. 3A,C). The pairwise difference values Vn/Vn + 1 of the internal reference genes (Fig. 3B,D) showed that V2/3 = 0.063 < 0.15 for proliferation and V2/3 = 0.084 < 0.15 for differentiation, indicating that the two reference genes were sufficient to accurately normalize the expression of the target genes.

#### NormFinder analysis

**Figure 4:**
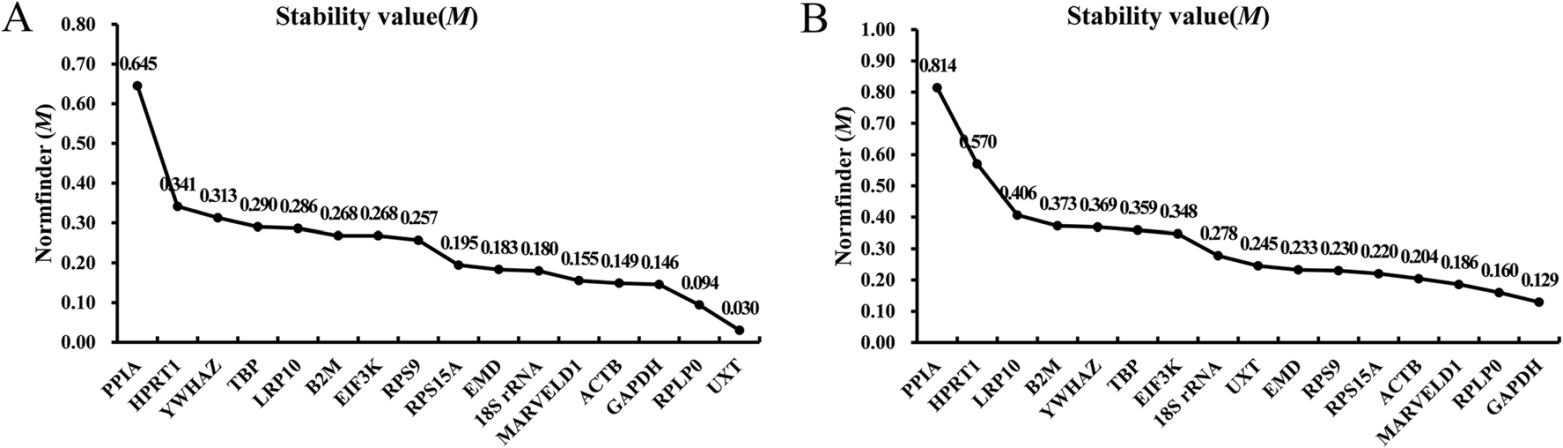
Expression stability of candidate reference genes as analyzed using the NormFinder program. (**A**) Stability of candidate reference genes in bovine SMSCs during proliferation. (**B**) Stability of candidate reference genes in induced differentiation of bovine SMSCs.

NormFinder software calculates arbitrary stability values and standard errors of the reference genes while taking into account the intra- and extra-group variation of each internal reference gene. According to the results given by NormFinder (Fig. 4), the stability value, M, of the reference genes ranged from 0.030 to 0.645 in the proliferation stage and from 0.129 to 0.814 in the differentiation stage, all of which reflected high stability. The two most stable internal reference genes screened by the software in the proliferation assay were *UXT* (0.030) and *RPLP0* (0.094) (Fig. 4A). In the differentiation assay, the two most stable internal reference genes were *GAPDH* (0.129) and *RPLP0* (0.160) (Fig. 4B). These results differed slightly from those of the GeNorm software analysis. In the proliferation and differentiation assays, the software identified the least stable gene as *PPIA* (0.645, 0.663), which is consistent with GeNorm software analysis.

#### Bestkeeper analysis

Bestkeeper software estimates the stability of a reference gene based on the variance and provides information about the coefficient of variation and correlation coefficients between samples, as the coefficient of variation (CV) ± standard deviation (SD). According to the stability rankings given by the software, the SD values of the internal reference genes during proliferation of SMSCs were between 0.54 and 0.91 (except for *PPIA* at 1.25). The two most stable genes screened by the software were *EIF3K* (1.51 ± 0.35) and *RPS15A* (2.01 ± 0.39) (Table 1). At the differentiation stage of SMSCs, the stability ranking results given by the software showed that twelve of the sixteen internal reference genes had SD values between 0.47 and 0.92, and four internal reference genes were > 1.00. The two most stable genes screened by the software were *TBP* (1.67 ± 0.47) and *EMD* (2.35 ± 0.61) (Table 2). The results were slightly different from those obtained in the GeNorm and NormFinder software analyses. The least stable internal reference gene screened by the software was *PPIA* (5.75 ± 1.25, 7.62 ± 1.88) (Table 1, 2) in proliferation and differentiation stages, which was consistent with the results obtained from GeNorm and NormFinder software.

**Table 1:** Stability of expression of candidate reference genes in bovine SMSCs at the proliferation stage estimated by Bestkeeper algorithm.

Gene	Coefficient of variation (CV)	Standard deviation (SD)	Rank
*18S rRNA*	3.75	0.54	9
*ACTB*	3.52	0.63	12
*B2M*	3.70	0.86	14
*EIF3K*	1.51	0.35	1
*EMD*	1.69	0.42	4
*GAPDH*	2.09	0.42	5
*HPRT1*	3.60	0.91	15
*LRP10*	1.57	0.40	3
*MARVELD1*	2.45	0.60	11
*PPIA*	5.75	1.25	16
*RPLP0*	2.90	0.59	10
*RPS15A*	2.01	0.39	2
*RPS9*	2.04	0.42	6
*TBP*	1.82	0.51	8
*UXT*	1.73	0.49	7
*YWHAZ*	3.55	0.82	13

**Table 2:** Expression stability of candidate reference genes in bovine SMSCs after induction of differentiation estimated by Bestkeeper algorithm.

Genes	Coefficient of variation (CV)	Standard deviation (SD)	Rank
*18S rRNA*	5.16	0.82	8
*ACTB*	4.62	0.92	12
*B2M*	4.27	1.04	13
*EIF3K*	2.72	0.65	4
*EMD*	2.35	0.61	2
*GAPDH*	4.06	0.88	11
*HPRT1*	5.64	1.56	15
*LRP10*	2.40	0.62	3
*MARVELD1*	3.23	0.83	9
*PPIA*	7.62	1.88	16
*RPLP0*	4.12	0.87	10
*RPS15A*	3.71	0.76	6
*RPS9*	3.57	0.78	7
*TBP*	1.67	0.47	1
*UXT*	2.54	0.75	5
*YWHAZ*	4.83	1.22	14

### Comprehensive analysis of candidate internal reference genes

To combine the results of analysis by the three procedures, the stability ranks of the candidate reference genes were calculated and ranked by geometric mean for both proliferation and induced differentiation stages of bovine SMSCs (Fig. 3). In the proliferation stage, the comprehensive stability rankings showed that *GAPDH* (2.47) and *RPS15A* (2.52) were the two most stable internal reference genes (Table 3). After induction differentiation, the comprehensive stability ranking results showed that *RPS15A* (3.11) and *RPS9* (3.43) were the two most stable internal reference genes (Table 4). The least stable reference gene in the comprehensive analysis of both proliferation and differentiation was *PPIA* (Tables 3 and 4). Therefore, *GAPDH* and *RPS15A* were the best reference gene combination to normalize the expression of target genes during proliferation, and *RPS15A* and *RPS9* were the best reference gene combination to normalize the expression of target genes during the induction of differentiation.

**Table 3:** Stability classes of candidate reference genes in the proliferation of bovine SMSCs analyzed by a combination of the three algorithms.

Gene	Program			Mean rank	Comprehensive rank
	GeNorm	NormFinde	BestKeeper		
*18S rRNA*	7	6	9	7.23	8
*ACTB*	8	4	12	7.27	9
*B2M*	13	11	14	12.60	13
*EIF3K*	9	10	1	4.48	5
*EMD*	5	7	4	5.19	6
*GAPDH*	1	3	5	2.47	1
*HPRT1*	15	15	15	15.00	15
*LRP10*	11	12	3	7.34	10
*MARVELD1*	6	5	11	6.91	7
*PPIA*	16	16	16	16.00	16
*RPLP0*	3	2	10	3.91	4
*RPS15A*	1	8	2	2.52	2
*RPS9*	10	9	6	8.14	11
*TBP*	12	13	8	10.77	12
*UXT*	4	1	7	3.04	3
*YWHAZ*	14	14	13	13.66	14

**Table 4:** Stability ranking of candidate reference genes during induced differentiation of bovine SMSCs analyzed by the three combined algorithms.

Gene	Program			Mean rank	Comprehensive rank
	GeNorm	NormFinde	BestKeeper		
*18S rRNA*	11	9	8	9.25	12
*ACTB*	10	4	12	7.83	10
*B2M*	13	13	13	13.00	13
*EIF3K*	5	10	4	5.85	8
*EMD*	6	7	2	4.38	5
*GAPDH*	7	1	11	4.25	4
*HPRT1*	15	15	15	15.00	15
*LRP10*	12	14	3	7.96	11
*MARVELD1*	4	3	9	4.76	7
*PPIA*	16	16	16	16.00	16
*RPLP0*	3	2	10	3.91	3
*RPS15A*	1	5	6	3.11	1
*RPS9*	1	6	7	3.48	2
*TBP*	9	11	1	4.63	6
*UXT*	8	8	5	6.84	9
*YWHAZ*	14	12	14	13.30	14

### Expression validation of candidate internal reference genes

**Figure 5:**
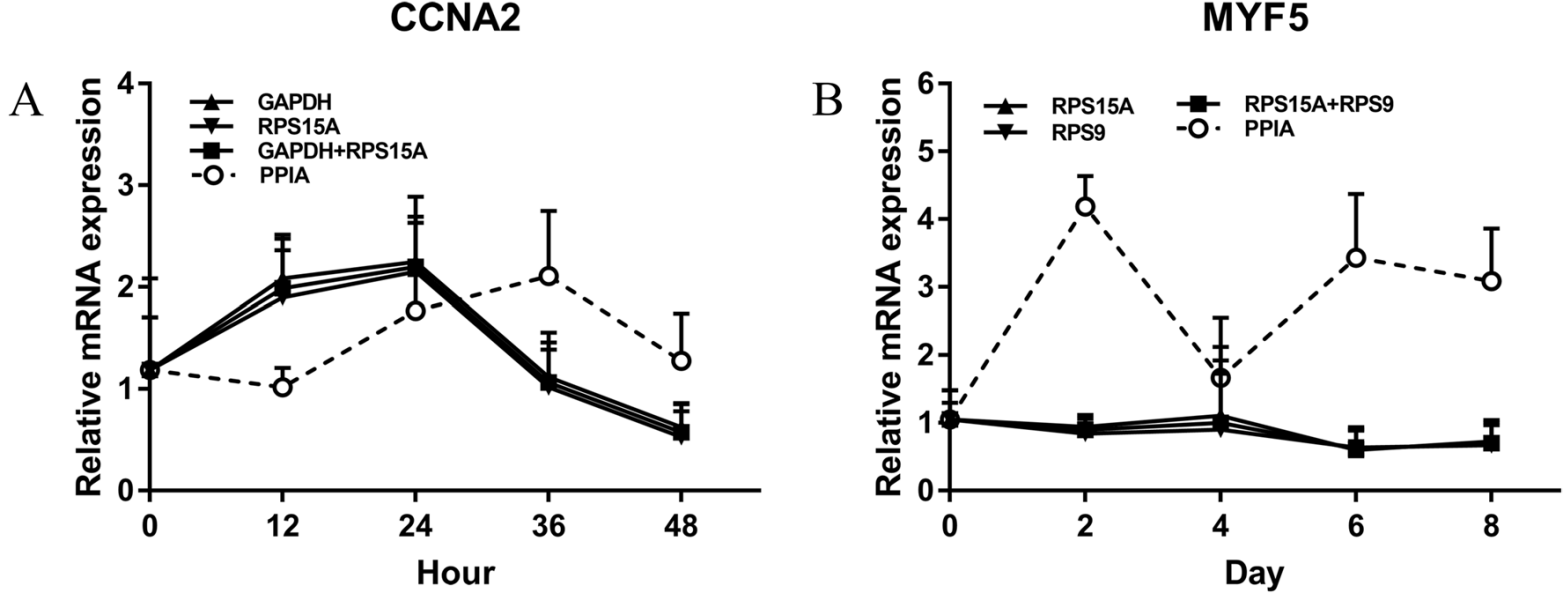
Effect of reference gene normalization on *CCNA2* and *MYF5* gene expression in bovine skeletal muscle-derived satellite cells. (**A**) Expression of *CCNA2* during proliferation was normalized to the geometric means of *GAPDH*, *RPS15A*, *PPIA* and *GAPDH* + *RPS15A*. (**B**) Expression of *MYF5* during the induced differentiation phase was normalized to the geometric means of *RPS15A*, *RPS9*, *PPIA*, and *RPS15A* + *RPS9*.

The effects of the most and least stable internal reference genes on gene expression during proliferation and induced differentiation of bovine SMSCs were investigated. The mRNA expression of the marker genes was similar when the two most stable reference genes were used alone or in combination, whereas the mRNA expression of the marker genes was different when the least stable reference gene was used (Fig. 5). *Cyclin A2* (*CCNA2*) is a ubiquitously expressed member of the cyclin family^14,15^. It plays an indispensable role in regulating the G1/S transition and in the process of mitosis through the activation of kinases^15^. *CCNA2* is frequently related to cell proliferation and usually considered as a marker of cell proliferation^15^. During the proliferation of bovine SMSCs, the mRNA expression of *CCNA2* first increased (0 h to 24 h) and then decreased (24 h to 48 h) when the geometric mean of *GAPDH* and *RPS15A* was used together or separately (Fig. 5). When *PPIA* was used as the correction factor, the mRNA expression of *CCNA2* decreased from 0 to 12 h, then increased from 12 to 36 h, and finally decreased from 36 to 48 h. Myogenic regulatory factor 5 (*MYF5*) is a key transcription factor that plays a central role in transcriptional regulation during muscle formation^16^. *MYF5* is implicated in the initial steps of myoblast differentiation^17^ and, therefore, is often used as a marker gene for differentiation of SMSCs. During the induced differentiation of bovine SMSCs, the mRNA expression of *MYF5* tended to decrease (0 d-2 d), then increase (2 d-4 d), then decrease (4 d-6 d), and finally increase (6 d-8 d), when the geometric means of *RPS15A* and *RPS9* were used as correction factors together or separately. When *PPIA* was used as a correction factor, the mRNA expression of *MYF5* was different from the former, showing an increase (0 d to 2 d), followed by a decrease (2 d to 4 d), then an increase (4 d to 6 d), and finally a decrease (6 d to 8 d). The trend was opposite to that when the two most stable reference genes were used alone or in combination and had a larger magnitude of change, peaking at 2 d with a 4.19-fold upregulation. It is clear that use of inappropriate reference genes may cause misinterpretation of target gene expression. Therefore, it is important to use the appropriate stable reference gene for relative gene expression.

## Discussion

Although qRT-PCR techniques have been widely used to elucidate gene expression in the proliferation and differentiation of bovine skeletal muscle-derived satellite cells in vitro, there is no unequivocal data on the most stable reference genes to use for gene expression measurement. In this study, we investigated the proliferation and differentiation of bovine skeletal muscle-derived satellite cells in vitro and compared their Ct values by qRT-PCR using sixteen commonly used reference genes, and then assessed the stability of Ct values using three commonly used algorithms (GeNorm, Normfinder and Bestkeeper). Our study found that *GAPDH* and *RPS15A* were the most stable reference genes in the proliferation of bovine SMSCs, while *RPS15A* and *RPS9* were the two most stable reference genes in the induction differentiation of bovine SMSCs. The least stable internal reference gene in proliferation and differentiation was *PPIA*. These reference genes validated under each experimental condition are a prerequisite for reliable assessment of gene expression by qRT-PCR, as the selection of inappropriate reference genes may yield inaccurate values or even erroneous or contradictory results^18–20^. To the best of our knowledge, this study is the first to validate the stability of reference genes in the proliferation and differentiation of bovine SMSCs in vitro.

This study showed that *GAPDH* was ranked 4th overall among sixteen candidate reference genes in the in vitro differentiation of bovine SMSCs and had good stability. It was also one of the most stable reference genes in the in vitro proliferation of bovine SMSCs. One study found that *GAPDH* was a stable reference gene for C2C12 myoblast differentiation, which is consistent with the present study^21,22^. Previous studies found that *GAPDH* was not suitable for the normalization of skeletal muscle development in cattle, and similar experimental results were obtained in pigs, goats and mice^10,23–25^. However, those experiments were conducted on the development of muscle tissue, whereas the present study on the proliferation and induction of differentiation of bovine primary skeletal muscle in vitro may have inconsistent results with *GAPDH* as an internal reference gene. In other tissue studies in cattle, *GAPDH* as a reference gene was stably expressed in buffalo oocytes, bovine endometrium and bovine blastocysts collected in winter and summer^26–28^, while it was expressed with low stability in bovine ovaries, corpus luteum and myometrium^29–31^, indicating that *GAPDH* had different stability as an internal reference gene in different tissues of cattle. The results of this study showed that *GAPDH* has good stability in both proliferation and induced differentiation of bovine SMSCs in vitro. This differs from studies of different tissues in cattle, suggesting that there may be differences in the selection of internal reference genes between in vitro cultures of cells and studies of tissues.

Our study also revealed that *RPS15A* was one of the most stable internal references in the proliferation of bovine SMSCs in vitro; *RPS15A* and *RPS9* were the two most stable RGs in the induction of differentiation of bovine SMSCs in vitro. This indicates that *RPS15A* has good stability in the proliferation or induced differentiation in vitro. *RPS15A* and *RPS9* belong to the ribosomal protein family. Several studies have been conducted with other bovine specimens to verify the stability of ribosomal protein family genes as internal reference genes under different experimental conditions. It has been shown that *RPS15A* and *RPS9* were the most stable internal reference genes in the Indian buffalo and bovine peripheral blood mononuclear cells^7^. *RPL15* was also the most stable internal reference in bovine oocytes collected in winter and summer^28^, while *RPL4* was the most stable internal reference gene in bovine bone marrow mesenchymal stem cell differentiation^32^. *RPS9* and *RPL19* in bovine maternal reproductive tissues and fetal tissues were found to be stably expressed^33^. In other species studies, *RPL13A* and *RPL4* were stable internal reference genes in long-term in vitro cultures of human bone marrow MSCs^20,34^. *RPL13* was the most stably expressed internal reference gene in visceral tissues of laying hens, broilers, and turkeys^35^. Similar to the results of the present study, these findings suggest the potential of ribosomal protein family genes to serve as broadly stable reference genes. However, it has also been shown that although ribosomal protein family genes were the most stably expressed, they may also not be truly stable reference genes^36^. The ribosomal protein family genes need to be tested to determine if they are stably expressed based on experimental data.

*PPIA* was the least stable internal reference gene in this study for both in vitro proliferation and induction of differentiation. It should be noted that the stability of *PPIA* in other tissues of cattle is different. *PPIA* was previously shown to be least stable in bovine adipose tissue, muscle, mammary gland and liver^37^, whereas it was stably expressed in oocytes of *Bos indicus* cattle, in bovine oocytes collected in winter and summer and bovine maternal reproductive and fetal tissues^28,33,38^. Similar results have been found in reference gene studies in other species, where *PPIA* was shown to be the least stable reference gene in the quadriceps muscle of energy-restricted mice^39^. However, *PPIA* was the most stable reference gene for gene expression in the longissimus dorsi (LD) muscle of postnatal Yorkshire pigs^10^. The differences in stability of reference genes were considered to be due to differences in experimental conditions, and verifying reference gene stability under each experimental condition was considered a necessary step before analyzing bovine gene expression by qRT-PCR^18,19,40^.

In this study, we performed an extensive literature search and selected sixteen candidate internal reference genes commonly used in cattle. Although we selected a large number of genes, these candidates may be limited, and there may be more stable internal reference genes that we did not select. The results of transcriptome sequencing to select the candidate reference gene may be more satisfactory, and this may be the direction of stable reference gene selection in the future. In our present study, we selected skeletal muscle-derived satellite cells from the longest dorsal muscle of Qinchuan cattle, however, different sites of skeletal muscle-derived satellite cells may show different results^7,41^.

In summary, this is the first time that the expression stability of a set of candidate reference genes was validated in vitro for studies of proliferation and induced differentiation of bovine skeletal muscle-derived satellite cells. Three different statistical calculations showed slight differences in the final ranking of reference genes; however, by combining the data, we determined the best combination of stably expressed and least stably expressed internal reference genes for proliferation and induced differentiation of bovine SMSCs under the present experimental conditions. Our results provide an important reference for the selection of stable internal reference genes for bovine gene expression by qRT-PCR analysis in future studies of in vitro proliferation and induced differentiation of bovine SMSCs.

## Conclusions

In summary, we evaluated the stability of sixteen reference genes in skeletal muscle-derived satellite cells during in vitro proliferation and induced differentiation using three assays (geNorm, NormFinder, and BestKeeper) to identify the most stable reference genes under different conditions. Our findings suggested that two reference genes were sufficient for accurate normalization in most conditions, although in some cases more than two reference genes may be required to accurately assess the changes in gene expression levels. Importantly, our data indicated that *GAPDH* and *RPS15A* were the most suitable reference genes during proliferation in skeletal muscle-derived satellite cells; and that *RPS15A* and *RPS9* were the most suitable reference genes during differentiation. The gene, *PPIA* was the most variable and least suitable for normalization of SMSCs during in vitro proliferation and induction of differentiation. These findings provide important data for selecting suitable reference genes in future studies. Our data provide guidelines for the selection of appropriate reference genes for studies on skeletal muscle development in mammals.

## Materials and Methods

### Ethical affirmation

In this study, three-day-old healthy Qinchuan beef cattle were used for myogenic cell isolation in accordance with the guidelines established by the Regulations on the Management of Laboratory Animal Affairs (Ministry of Science and Technology, China, 2004) and approved by the Institutional Animal Care and Use Committee (School of Animal Science and Technology, Northwest Agriculture and Forestry University). Cattles were raised under free-range conditions and samples were collected after euthanasia at the National Beef Cattle Improvement Center (Yangling, China). This study was carried out in compliance with the ARRIVE guidelines.

### Isolation and culture of bovine skeletal muscle-derived satellite cells (SMSCs)

SMSCs were obtained from three-day-old healthy Qinchuan beef cattle and isolated as previously described by Wang Yaning, et al.^42^. The cells were cultured in DMEM/F-12 (Gibco, Shanghai, China), supplemented with 20% FBS (Gibco, Shanghai, China) and 1% penicillin/streptomycin (Hyclone, Thermo-Fisher Scientific, Shanghai. China). When the confluence reached about 80%, the cells were seeded into six-well plates and in vitro proliferation of SMSCs was measured starting from 0 h at a confluence of about 50%. The cells were collected at 0, 12, 24, 36 and 48 h, with three biological replicates for each time point. To study induction of differentiation, SMSCs were grown in culture medium to 80% confluence in six-well plates, and on day-zero (0 d) the medium was changed to differentiation medium containing DMEM/F-12, 2% horse serum (Gibco, Shanghai, China) and 1% penicillin/streptomycin. The cells were collected at 0, 2 , 4 , 6 , and 8 d, with three biological replicates at each time point.

### Immunofluorescence assay for myocyte-specific protein

SMSCs were cultured in 12-well plates, fixed with 4% paraformaldehyde for 15 min at room temperature, washed with PBS, permeabilized with 0.2% Triton X-100 for 15 min and then incubated in 10% (vol/vol) normal donkey serum/1% BSA (Sigma) /0.3 M glycine (Sigma) for 1 h to block non-specific protein–protein interactions at room temperature. For immunofluorescence, the cells were incubated with the primary antibody (diluted in 10% normal donkey serum/1% BSA/0.3 M glycine) overnight at 4˚C. The cells were then washed with PBS and incubated with secondary antibody at 37˚C for 1 h (protected from light). The nuclei were stained with DAPI (Sigma) at room temperature for 15 min (protected from light). The antibodies used were as follows: anti-MYHC (1:300, GeneTex), and donkey anti-mouse IgG H&L (AlexaFluor1 555) (1:1000, Abcam). DAPI was used at a concentration of 1 μg/ml. Immunofluorescence images were obtained using an Evos-fl-auto2 microscopy imaging system (Thermo Scientific, USA).

### RNA extraction and reverse transcription to cDNA

Total RNA was extracted from the third cell passage of bovine SMSCs using a kit containing TRIzol (Takara) according to instructions. The quality and concentration of the RNA were determined by electrophoresis of aliquots on a 1% agarose gel and measuring A_260_/A_280_ with a microplate reader. Using the PrimeScript RT kit with gDNA Eraser (Perfect Real Time; Takara), 1 µg of total RNA was reverse transcribed to cDNA and stored at -80 °C for backup.

### Primer design

The primer sequences of relevant reference genes in bovine skeletal muscle cells from previous studies were checked by the Primer-Blast program (NCBI tools). The primers with good specificity were used directly, while those with poor specificity were redesigned using Primer Premier 5, according to primer length, annealing temperature, base composition and 3'-end stability; specificity of the primers was checked using the Primer-Blast program (NCBI tools) to ensure primer specificity (Table 5).

**Table 5:** Primer sequences for qRT-PCR.

Gene symbol	Gene name	GenBank Accession #	Primer Sequences (5' → 3')	Ampli-con (bp)	References
*GAPDH*	glyceraldehyde-3-phosphate dehydrogenase	NM_001034034.2	F: ACACCCTCAAGATTGTCAGCAA	102	^43^
			R: TCATAAGTCCCTCCACGATGC		
*ACTB*	β-actin	NM_173979.3	F:CATCGGCAATGAGCGGTTCC	147	This study
			R:ACCGTGTTGGCGTAGAGGTC		
*PPIA*	peptidylprolyl isomerase A	NM_178320.2	F:TCAACCCCACCGTGTTCTTC	189	This study
			R:CACCCTGGCACATAAATCCC		
*LRP10*	LDL receptor-related protein 10	NM_001100371.1	F:CCATTCGCACCCAGGAGTACA	167	This study
			R:GCCCAGAACAGAGTTATCATTAGG		
*HPRT1*	hypoxanthine phosphoribosyl-transferase I	NM_001034035.2	F:AACGACCAGTCAACAGGCGA	191	This study
			R:CGAGGGGTCCTTTTCATCAG		
*YWHAZ*	tyrosine 3-monooxygenase /tryptophan 5-monooxygenase activation protein, zeta polypeptide	NM_174814.2	F:GTGGACCAGTCACAGCAAGC	134	This study
			R:TCAGGGGAGTTCAGAATCTCATAA		
*B2M*	Beta-2-microglobulin	NM_173893.3	F:CCACCAGAAGATGGAAAGCC	171	^44^
			R:AGTGAACTCAGCGTGGGACA		
*TBP*	TATA box-binding protein Tyrosine	NM_001075742.1	F:AACAGCCTCCCACCCTATGC	71	^28^
			R:AAGATAGGGATTCCAGGAGTCATG		
*EIF3K*	Eukaryotic translation initiation factor 3 K	NM_001034489.2	F:CTGGCTGAGATGCTCGGGG	136	^45^
			R:CCACGATGTTCTTGGGCTTTAT		
*RPS9*	Ribosomal protein S9	NM_001101152.2	F:CGAAGGTAATGCCCTGTTGC	204	This study
			R:TGCTTGCGGACCCTGATGT		
*UXT*	Ubiquitously expressed prefoldin-like chaperone	NM_001037471.2	F:TCATGGCGACGCCCCCTAAAC	70	^46^
			R:AAAGCCTCGTAGCGCAGCACT		
*18 s rRNA*	18S ribosomal RNA	NR_036642.1	F:CCTGCGGCTTAATTTGACTC	118	This study
			R:AACTAAGAACGGCCATGCAC		
*RPLP0*	Ribosomal protein LP0	NM_001012682.1	F:TGGTTACCCAACCGTCGCATCTGTA	142	^47^
			R:CACAAAGGCAGATGGATCAGCCAAG		
*MARVELD1*	MARVEL domain‐containing 1	NM_001101262.1	F: GGCCAGCTGTAAGATCATCACA	100	^23^
			R: TCTGATCACAGACAGAGCACCAT		
*EMD*	emerin	NM_203361.1	F:GCCCTCAGCTTCACTCTCAGA	100	^23^
			R:GAGGCGTTCCCGATCCTT		
*RPS15A*	Ribosomal protein S15A	NM_001037443.2	F:TCAGCCCTAGATTTGATGTGC	148	This study
			R:TTCCCTCCTGTATGTTTTCGTC		
*CCNA2*	Cyclin A2	NM_001075123.1	F:AGTATTTGCCGTCAGTTATCGC	184	This study
			R:CTTATTGACTGTTGTGCGTGCT		
*MYF5*	Myogenic factor 5	NM_174116.1	F:CCAGCACCGATTCTCAACCT	151	This study
			R:CAGGTTGTCTTGCTTTGGGG		

### Standard curve construction of qRT-PCR primers

A total of sixteen genes, including *GAPDH, ACTB, PPIA, LRP10, HPRT1, YWHAZ, B2M, TBP, EIF3K, RPS9, UXT, 18S rRNA, RPLP0, MARVELD, EMD* and *RPS15A*, were selected as candidate internal reference genes in this study. The primers were synthesized by Chengdu Prime Biotechnology Co., Ltd, and the cDNA template was diluted by 1, 5^–1^, 5^–2^, 5^–threefold^ for qRT-PCR. The standard curve was plotted as the logarithm of the sample concentration on the x-axis and the ct value on the y-axia. The amplification efficiency was calculated according to the formula: E = 10^(-1/slope)^ − 1.

### Quantitative real-time PCR (qRT-PCR)

The cDNA stored at − 80 °C was diluted tenfold and qRT-PCR was performed using SYBR green real-time PCR master mix (Takara) in a CFX96 BIO-RAD thermocycler (USA). The final reaction volume of 25 µl contained 12.5 µl of SYBR Premix Ex Taq II (Tli RNaseH Plus) (2 ×), 1 µl each of forward and reverse primers (final primer concentration 0.4 µM), 2 µL of cDNA and 8.5 µL dd H_2_O. Thermocycling conditions were: denaturation at 95 °C for 5 min followed by forty amplification cycles with denaturing at 95 °C for 30 s, 60 °C for 30 s and 72 °C 30 s.

### Stability analysis of reference genes

The expression stability of selected reference genes was evaluated using three programs: GeNorm^11^, Normfinder^12^, and Bestkeeper^13^, following the developer's instructions. GeNorm was also used to calculate pairwise variations (V/V values) and to determine the minimum number of reference genes required for accurate normalization. The geometric mean of the ranking values of the internal reference genes in different software was used for integrated stability analysis to obtain a reliable agreement.

### Validation of reference gene expression

The stability of the tested reference genes was verified by measuring the expression of *CCNA2* at different times during proliferation and the expression of *MYF5* at different times during the differentiation of bovine SMSCs in vitro. The expression of *CCNA2*, *MYF5* was normalized using the most stable candidate reference gene group and the least stable candidate reference gene.

## Supplementary Information

Supplementary Information 1.

Supplementary Information 2.
