# Supplementary material for: Screening and validation of reference genes for qRT-PCR of bovine skeletal muscle-derived satellite cells
Source: Sci Rep. 2022 Apr 5;12:5653. doi: 10.1038/s41598-022-09476-3 (PMC8983775; doi:10.1038/s41598-022-09476-3)
Supplement: Supplementary file 1 — Supplementary Information 1. [file 41598_2022_9476_MOESM1_ESM.docx]

**Supplementary Figures**


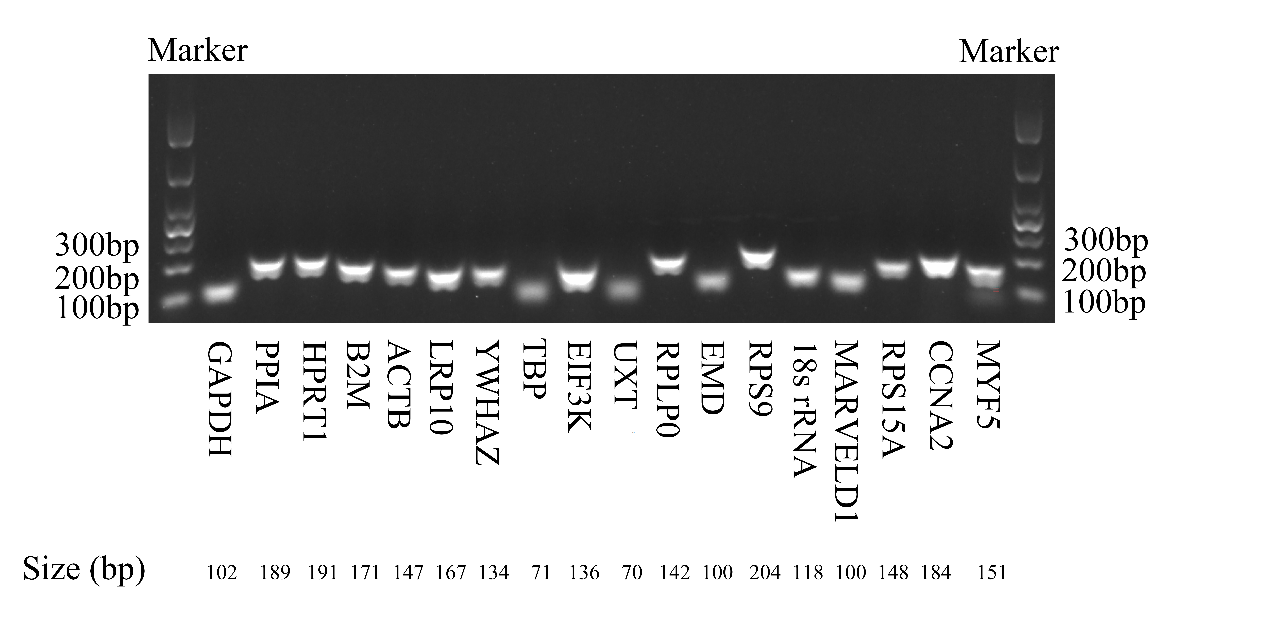


**Figure S1.** Agarose gel electrophoresis identification of gene-specific primers of reference genes for qPCR.


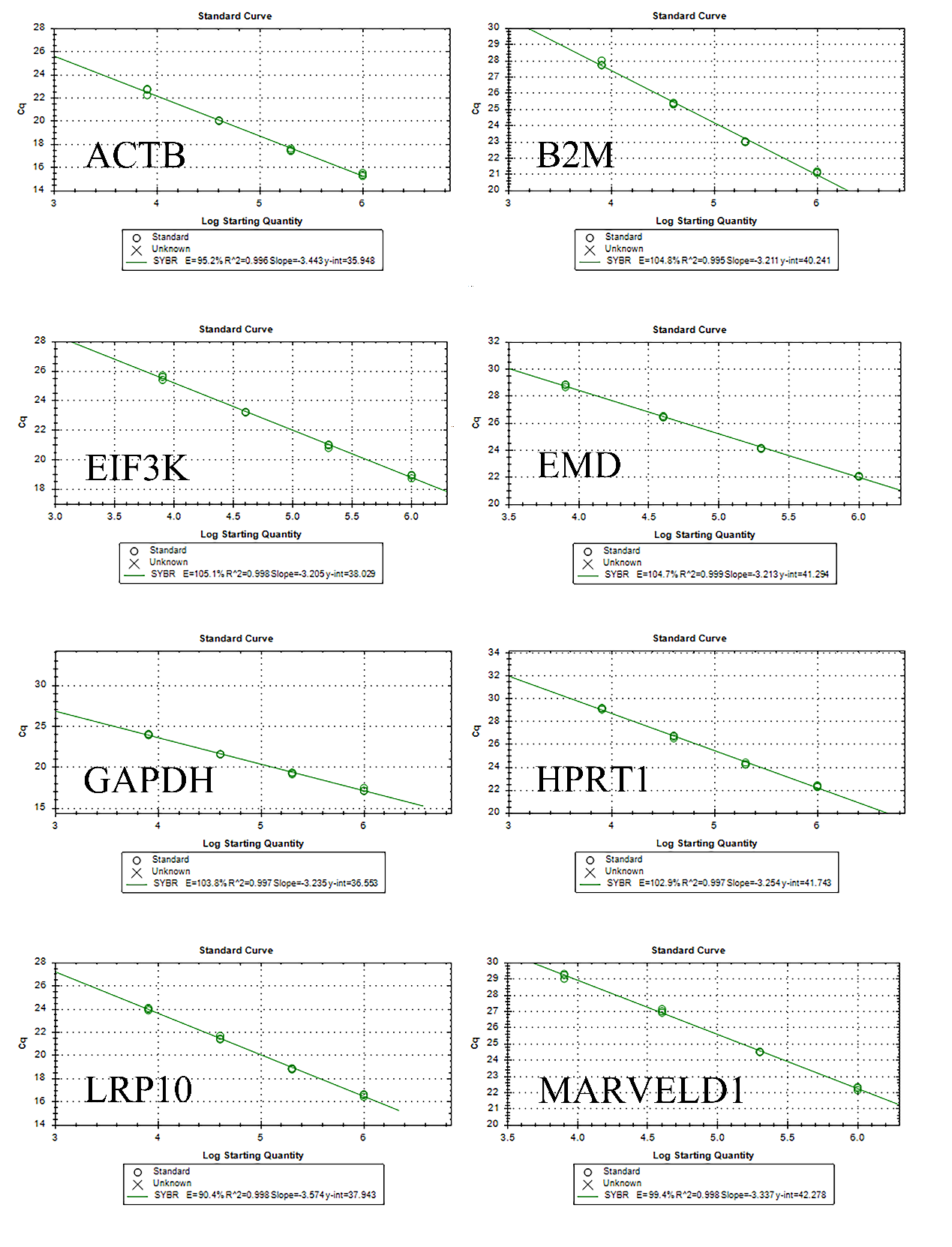


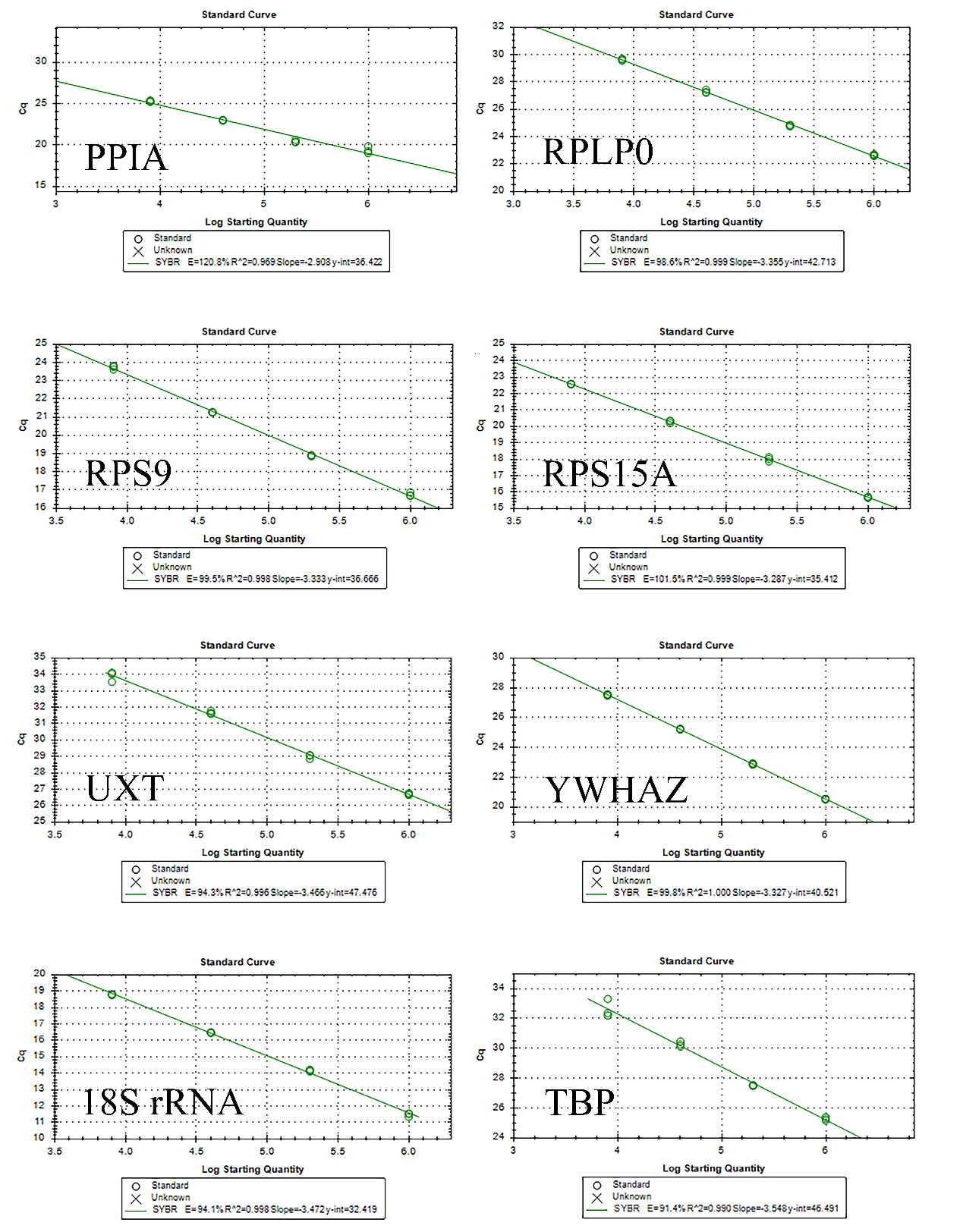


**Figure S2.** Standard curves of candidate reference genes for qRT-PCR.


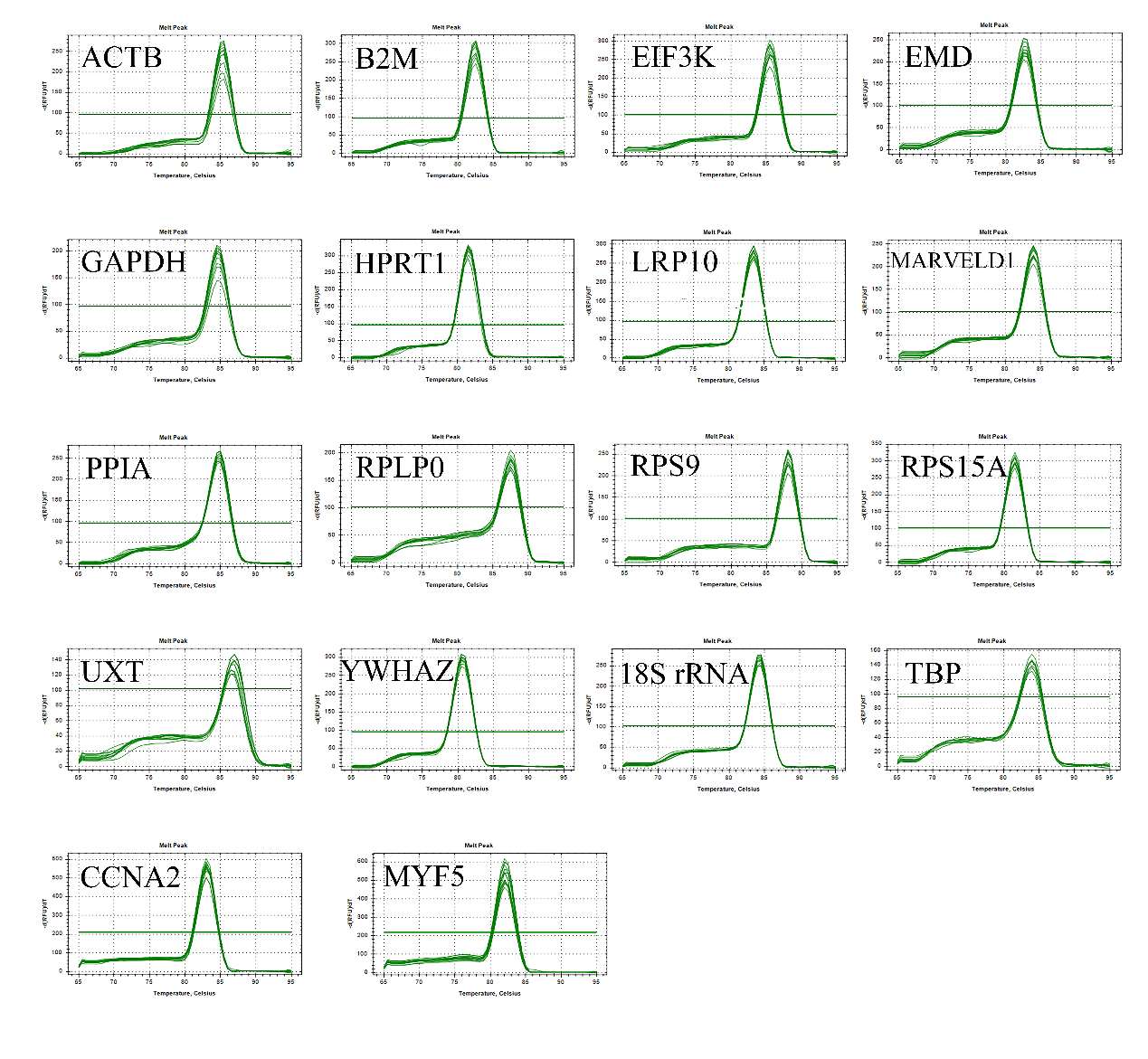


**Figure S3.** Melting curves of sixteen candidate reference genes and two target genes, obtained by qRT-PCR.
